# Draft genome sequence of *Taiwanofungus gaoligongensis* strain YAF008 isolated from Gaoligong Mountain

**DOI:** 10.1128/mra.00340-24

**Published:** 2024-09-09

**Authors:** Yuanduo Bao, Xiaolong Yuan, Zihong Chen, Yuming Yang, Yuan Zheng, Yi Wang

**Affiliations:** 1 College of Forestry, Southwest Forestry University, Kunming, China; 2 Yunnan Key Laboratory of Biodiversity of Gaoligong Mountain, Yunnan Academy of Forestry and Grassland, Kunming, China; 3 Yunnan Key Laboratory for Fungal Diversity and Green Development, Kunming, China; 4 College of Resources and Environment, Baoshan University, Baoshan, China; Rochester Institute of Technology, Rochester, New York, USA

**Keywords:** *Taiwanofungus gaoligongensis*, genome, nanopore sequencing

## Abstract

The draft genome sequence of *Taiwanofungus gaoligongensis* YAF008 was reported. The genome size of *T. gaoligongensis* YAF008 was 34.7M bp with 50.72% GC content. The genome resource will support future research into potential secondary metabolite diversity of this fungus.

## ANNOUNCEMENT

*Taiwanofungus camphoratus* (syn. *Antrodia cinnamomea*, *Antrodia camphorata*) is a valuable edible and medicinal fungus (1), belonging to Basidiomycota, Polyporales, Taiwanofungaceae, Taiwanofungus. *T. camphoratus* can produce a variety of active substances, such as polysaccharides, triterpenoids, quinone derivatives, and benzene derivatives (2–4). Studies have shown that these compounds have anti-cancer (5), anti-tumor (6), anti-oxidation (7), anti-inflammation (8), and liver protection (9) properties. In this study, we report the genome of *Taiwanofungus gaoligongensis* from Gaoligong Mountain.

The *T. gaoligongensis* strain YAF008 was isolated from the fallen bark of camphor tree. The isolation process is as follows. The bark was collected from Gaoligong Mountain, Yunnan Province, China (24° 73′ 14″ N, 99° 18′ 24″ E) in 2018. The bark was washed by soaking it in tap water for 24 hours to remove surface impurities and spores and then was washed with sterile water. The bark was ground by a tissue grinder. Homogenization buffer was obtained by mixing ground thallus and sterile water. Finally, the homogenized buffer of bark was diluted and spread onto a solid Malt-Yeast medium (Difco, Lawrence, USA) with 100 µg/mL ampicillin, kanamycin, and streptomycin. After 15 days of incubation (28°C), a single colony was transferred in new medium. One single colony YAF008 was identified as *T. gaoligongensis* by ITS. The ITS sequence was deposited in GenBank with the accession number OR681872. *T. gaoligongensis* strain YAF008 was deposited in the China Center for Type Culture Collection (deposit number: CCTCC M 20232425).

The mycelia of *T. gaoligongensis* strain YAF008 were harvested for DNA extraction by filter paper after cultivation in Malt-Yeast liquid medium at 28°C for 25 days on a rotary shaker at 150 rpm. Genomic DNA was extracted using a DNeasy minikit (Qiagen, Valencia, CA, USA) following the manufacturer’s instructions. The library preparation for the Illumina NovaSeq platform was performed using the TruSeq DNA Sample Prep Kit (New England Biolabs, USA) and that for the Nanopore PromethION platform was executed according to the ligation sequencing SQK-LSK110 kit (Oxford Nanopore Technologies, UK). Oxford Nanopore GUPPY (version 0.3.0) was used to filter the longer reads and quality reads from the 1.2 million reads obtained (9.19 Gbp) with a N50 of 19,690 bp. Default parameters were used for all software unless otherwise specified. The filtered Nanopore reads were assembled using NECAT (10). Racon (version:1.4.11) software was used to perform two rounds of error correction based on the third-generation sequencing data (11). For Illumina short reads, quality control was performed with AdapterRemoval version 2 (12). Pilon package version 1.23 (13) was used to correct the base errors of the assembled contigs by using the Illumina short reads. The genome assembly size of *T. gaoligongensis* was 34,703,244 bp with 19 scaffolds and the GC content was 50.72% (Table 1). The genome completeness was assessed using BUSCO v5.2.2 (14) with the OrthoDBfungi v10 (fungi_odb10) database, showing a completeness score of 96.6%.

**TABLE 1:** Genome sequencing statistics

Parameter	Value
Illumina sequencing	
Raw reads	
Reads number	36,300,454
Total bases (bp)	5,445,068,100
Nanopore sequencing	
Total sequence number	1,220,018
Total base	9,194,795,196
Genome assembly	
Total base	34,703,244
Number of scaffolds	19
Max sequence length	4,247,058 bp
N50	2,343,139 bp
N90	1,499,237 bp
GC_content (%)	50.72
Genome assembly assessment	
Complete BUSCOs	96.60%
Complete and single-copy BUSCOs	95.80%
Complete and duplicated BUSCOs	0.80%

## Data Availability

This study project is available under NCBI BioProject accession number PRJNA1051983 and BioSample accession number SAMN38809687. The complete genome assembly of *T. gaoligongensis* strain YAF008 is available under GenBank accession number JAZIAZ000000000. https://www.ncbi.nlm.nih.gov/datasets/genome/GCA_037127245.1/
